# Meta-Analysis of the Effect of Traditional Chinese Medicine Compounds Combined with Standard Western Medicine for the Treatment of Diabetes Mellitus Complicated by Coronary Heart Disease

**DOI:** 10.1155/2021/5515142

**Published:** 2021-06-18

**Authors:** Mengqi Yang, Min Cheng, Min Wang, Zhishu Tang, Zhongxing Song, Chunli Cui, Yanru Liu, Zhen Zhang

**Affiliations:** ^1^Shaanxi University of Chinese Medicine, Xianyang 712046, China; ^2^Co-construction Collaborative Innovation Center for Chinese Medicine Resources Industrialization By Shaanxi and Education Ministry, Xianyang 712046, China; ^3^State Key Laboratory of Research and Development of Characteristic Qin Medicine Resources (Cultivation), Xianyang 712046, China; ^4^Chinese Medicine Industry Research Institute, Xianyang 712046, China; ^5^Shangluo University, Shangluo 726000, China; ^6^The Second Affiliated Hospital, Shaanxi University of Chinese Medicine, Xianyang 712046, China

## Abstract

This study aimed to systematically evaluate the clinical efficacy and safety of traditional Chinese medicine (TCM) compounds combined with standard treatments for diabetes mellitus (DM) complicated by coronary heart disease (CHD). We performed a systematic and comprehensive search of the China Knowledge Network, WanFang, WeiPu, PubMed, and Web of Science, including Chinese and English articles, for randomized controlled trials (RCTs) assessing the use of Chinese herbal compounds for the treatment of DM complicated by CHD published before June 1, 2020. The literature was screened according to standard criteria. Risk assessment, based on the Jadad scale, was performed using the Review Manager5.3 software for meta-analysis. In total, 23 articles were selected, including 2405 cases. The meta-analysis showed that the combination of standard treatments with TCM compounds significantly improved the overall treatment efficacy for DM complicated by CHD (OR(odds ratio) = 4.39; 95% confidence interval (95% CI), 3.30–5.84; *P* < 0.0001), fasting blood glucose level (mean difference (MD) = −1.04; 95% CI, −1.96 to −0.13; *P*=0.03), total cholesterol level (MD = −1.16; 95% CI, −1.48 to −0.83; *P* < 0.0001), triglyceride (MD = −0.46; 95% CI, −0.62 to −0.29; *P* < 0.0001), low-density lipoprotein level (MD = −0.57; 95% CI, −0.87 to −0.27; *P*=0.0002), high-density lipoprotein level (MD = 0.19; 95% CI, 0.12 to 0.26; *P*=0.02), and electrocardiogram (OR = 4.20; 95% CI, 3.15 to 8.18; *P* < 0.0001). In contrast, there was no improvement of 2-hour postprandial glucose level (MD = −1.03; 95% CI, −2.14 to 0.08; *P*=0.07), or adverse reactions (OR = 0.53; 95% CI, 0.19 to 5.50; *P*=0.21). We concluded that the combined therapy has some benefits in treating DM complicated by CHD. However, these results should be confirmed by further referenced evidence, high risk assessment, and lower publication bias.

## 1. Introduction

Diabetes mellitus (DM) is a metabolic disorder characterized by hyperglycemia [1]. Its etiology is complex and related complications severely threaten human health. Both coronary heart disease (CHD) and DM are common clinical conditions. Coronary atherosclerotic heart disease is referred to as coronary artery disease, which leads to stenosis or occlusion of the artery lumen and eventually to myocardial ischemia [2]. Studies have shown that type 2 DM (T2DM) is associated with higher risk of CHD [3]. The pathological basis of CHD is myocardial ischemia, hypoxia, or necrosis caused by coronary atherosclerosis. DM is one of the risk factors for premature arteriosclerosis and can further contribute to the deterioration of CHD. Data showed that diabetic patients are four times more likely to develop cardiovascular disease than nondiabetic patients [4]. DM complicated by CHD often leads to multiple organ damage, seriously reducing the quality of life of the patients, and it can even be life threatening [5]. Clinical treatment of DM complicated by CHD mainly aims at improving myocardial blood supply and controlling blood glucose through the use of antiplatelet aggregation, anticoagulation, antiperoxidation, antihypertension, blood lipid regulation, thrombolysis, bypass grafting, and arterial interventional therapy. However, these strategies require long-term therapy, only have a single target, and associated with poor patient compliance with the drug regimen, adverse drug reactions, and poor prognosis, which leads to poor overall clinical efficacy [6]. In traditional Chinese medicine (TCM) theory, CHD and DM are classified as “chest arthralgia”, “heartache,” and “thirst elimination” [7].

The main clinical manifestation of CHD is angina pectoris, often accompanied by panic, shortness of breath, and fatigue. Ancient medical books recorded a lot of prescriptions for the treatment of chest arthralgia, including Wenyang Ton gmai, Yiqi Shengxue, Yangyin Shengmai, and other conventional prescriptions. TCM diagnosis criteria of DM is lung heat injury and fire-flourishing Yin deficiency, often calling for Xiaoke Fang and Qingwei San dialectical treatments [8]. Recently, the scientific literature reported that TCM compounds can exert certain therapeutic effects on DM and CHD [9]. A study by Zhang et al. [10] showed that Western medicine basic therapy supplemented by Yiqi Yangyin Huoxue decoction provides a better treatment for DM complicated by CHD than Western medicine used alone, and it could increase the curative effects of Western medicine. Another study by Zou [11, 12] showed that the addition of Yixinshu capsules can relieve symptoms in patients suffering from T2DM-related CHD angina pectoris, enhance exercise tolerance, reduce blood glucose level, and the capsule is safe, effective, and easily tolerated. Finally, addition of Pingxiaotongxin capsules added to standard treatment can also alleviate patient symptoms and reduce their blood glucose level. However, there is still insufficient evidence supporting the use of these TCM compounds. To fill this gap, we sought to provide statistics-based evidence as reference for the clinical application of TCM by performing a systematic meta-analysis comparing the efficacy of Western standard therapy used alone (control group) or in combination with TCM compounds (observation group) for the treatment of DM complicated by CHD.

## 2. Methods

### 2.1. Inclusion Criteria

The patients included in this study met the diagnostic criteria for CHD established by the Ministry of Health of the People's Republic of China and the diagnostic criteria for DM published by the World Health Organization in 2019. Only publications in English or Chinese were included. The included study's indicators of efficacy included at least total efficiency or blood glucose levels.

### 2.2. Exclusion Criteria

Excluded documents included literature reviews, meta-analyses, studies in animal models, duplicate publication, and retrospective studies, such as clinical studies designed without a control group or case reports.

### 2.3. Intervention

For inclusion of a study, the control group had to be treated with standard Western medicine protocol, and the observation group had to be treated with the same standard Western medicine protocol as the control group, in combination with TCM compounds. The specific dosage and time of administration were not clearly defined.

### 2.4. Evaluation Indicators

The clinical indicators included total effective rate, levels of fasting blood glucose, postprandial 2-hour plasma glucose, total cholesterol, triglycerides, high-density lipoprotein cholesterol, and low-density lipoprotein cholesterol, as well as frequency of angina attacks and the duration of angina pectoris pain.

### 2.5. Search Strategies

We searched the China National Knowledge Infrastructure (CNKI), WanFang, the Cochrane Library, and CBM (documents collected from January 2000 to February 2020) on traditional Chinese medicine compounds combined with standard Western medicine for the treatment of DM complicated by coronary heart disease. The language of the text was limited to Chinese and English. Theme for database searching were “Chinese medicine compound,” “DM with coronary heart disease,” and “randomized control”.

### 2.6. Data Extraction and Risk Assessment

In total, 23 articles were selected and cross-checked by two researchers. The controversial content or the score of the article was determined by one-third person after careful assessment. Collected information included the author names, year of publication, the numbers of cases, methodological characteristics, outcome measures, adverse events, and follow-up records. These data were separately collected and cross-checked by two researchers. The controversial content was determined by one-third person after careful assessment. Risk assessment was based on the Jadad scale. The specific points for the evaluation included randomization scheme (2 points), blind mode (2 points), and exit and missing visit (1 point). Based on the score, the articles were classified as low quality (0–2 points), medium quality (3–4 points), and high quality (5 points).

### 2.7. Statistical Analysis

The meta-analysis was performed using the Cochrane Collaboration's software RevMan 5.3. Heterogeneity was assessed by means of *I*^2^ statistics. The fixed effect model was used to analyze data with low heterogeneity. The random effects model was used to analyze the data with *I*^2^ >50%, representing high heterogeneity. Outcome measures, such as total efficiency rate (TER) and adverse events (AEs) as dichotomous variables, were analyzed using odds ratio (OR) values with 95% confidence interval (95% CI). Continuous data are presented as weighted mean difference (MD) with a 95% CI. Funnel plots were used to analyze publication bias.

## 3. Results

### 3.1. Literature Retrieval

**Figure 1:**
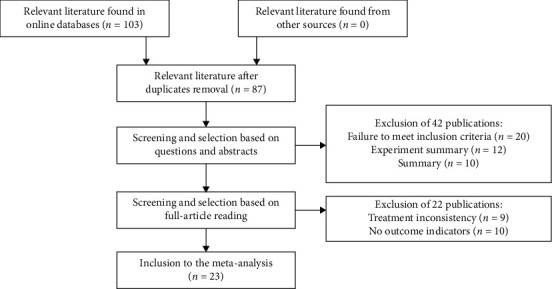
Search flow diagram.

In total, 23 randomized controlled trials (RCTs) were selected, including 2405 cases. The article screening process and results are shown in Figure 1.

### 3.2. Incorporation of the Basic Features of the Literature

Based on the above literature screening process, 23 RCTs were selected finally, gathering a total of 2405 samples, including 1219 were in the observation groups and 1186 were in the control groups. The demographic baseline indicators were not statistically different and comparable across the different studies. Specific information is reported in Table 1.

### 3.3. Document Evaluation

Based on the Jadad scale, the 23 articles were evaluated systematically from the aspects of “randomization mode,” “blind method strategy,” and “exit and missing visits.” Regarding the randomization mode, in the studies, Ye [17], Li and Shang [31], Guan and Shang [19], Xing et al. [18], Zhang and Zhang [16], Yan et al. [28], He [32], and Sun et al. [24] used the random number table method to perform a random grouping, which corresponds to a standard grouping mode and gave a 2-point assignment. Zhang [15] used a random allocation method, Zhang [13] used a random group method, and Zhang [27] used a file sequence all corresponding to “pseudorandomization” methods and that gave 0-point assignment. In the other selected articles [14, 20–23, 25, 26, 29–31, 33–35], no randomization method was specified, and 0 point were assigned. None of the selected studies had implemented a blinding procedure, and therefore, all scored 0. Studies that did not have any dropout or lost follow-up were assigned 1 point. Based on the above scoring, only 8 of the 23 selected articles reached a medium quality (3 points), whereas the other articles were of low quality (1 point). The overall article evidence level was low, and the error risk was high, as shown in Table 2.

### 3.4. Meta-Analysis

#### 3.4.1. Total Clinical Efficiency

**Figure 2:**
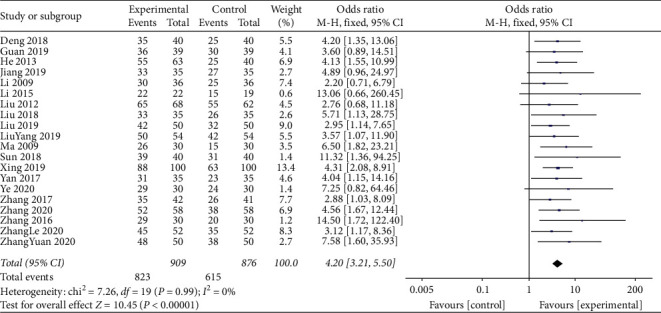
Meta-analysis of total clinical efficiency.

A total of 1785 participants were reported in 20 studies with no statistical between-study heterogeneity (*I*^2^ = 0%; *P*=0.09). Therefore, a meta-analysis of the data from these 20 studies was performed according to the fixed effect model. The results of the comparison between standard treatments alone and standard treatment combined with TCM compounds showed that the TCM compounds improved the overall clinical efficiency of treatment for DM complicated by CHD (OR = 4.20; 95% CI, 3.21–5.50; *P*=0.0001; Figure 2).

#### 3.4.2. Fasting Blood Glucose (FBG) Level

**Figure 3:**
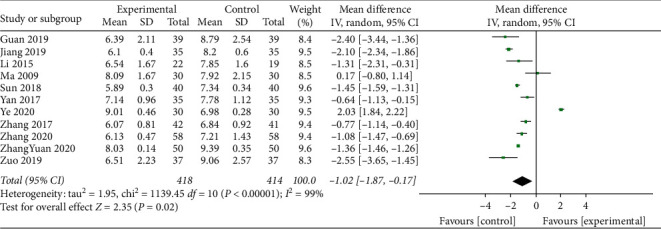
Meta-analysis of fasting blood glucose levels.

The FBG levels were reported for a total of 832 participants across 11 studies that were statistically heterogeneous (*I*^2^ = 99%; *P* < 0.001). Therefore, the meta-analysis of the data was performed using the random effects model. The results showed that addition of TCM compounds to standard treatments reduced the FBG levels (MD = −1.02; 95% CI, −1.87 to −0.17; *P*=0.02; Figure 3).

#### 3.4.3. Two-Hour Plasma Glucose (2hPG)

**Figure 4:**
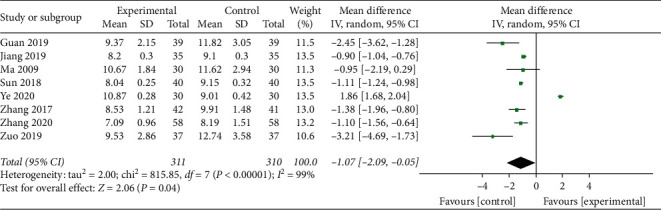
Two-hour plasma glucose (2hPG) levels.

The 2hPG levels were reported for a total of 621 participants across 8 studies that presented with statistical between-study heterogeneity (*I*^2^ = 99%; *P* < 0.001). Therefore, the meta-analysis of the data was performed using the random effects model. The comparison between standard treatments alone and standard treatments combined with TCM compounds showed that the addition of the TCM compounds to the therapy had no effect on 2hPG levels (MD = −1.07; 95% CI, −2.09 to −0.08; *P*=0.04; Figure 4).

#### 3.4.4. Total Cholesterol

**Figure 5:**
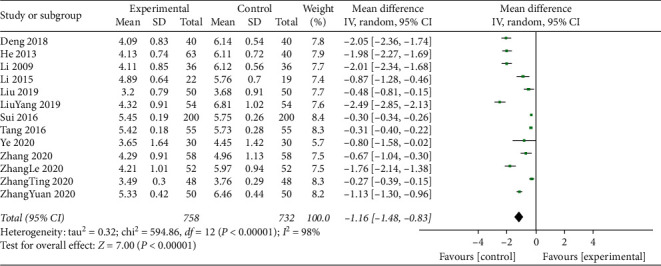
Meta-analysis of total cholesterol levels.

Total cholesterol levels were measured for a total of 1490 participants across 13 studies presented with statistical between-study heterogeneity (*I*^2^ = 98%; *P* < 0.001). Therefore, the meta-analysis of the data was performed using the random effects model. This analysis showed that TCM compounds combined with standard treatments ameliorated the total cholesterol levels of the patients suffering from DM complicated by CHD complications, compared with standard treatments used alone (MD = −1.16; 95% CI, −1.48 to −0.83; *P*=0.0001; Figure 5).

#### 3.4.5. Triglycerides

**Figure 6:**
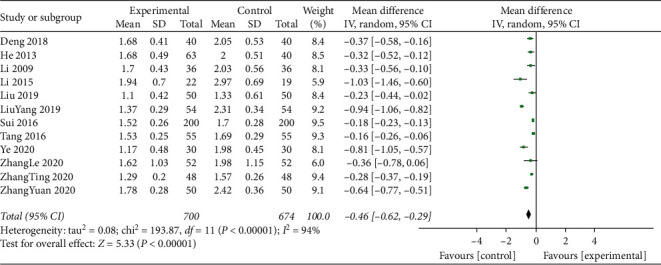
Meta-analysis of triglyceride levels.

Triglyceride levels were reported for a total of 1374 participants in 12 studies. The statistical heterogeneity of the results from these studies (*I*^2^ = 94%; *P* < 0.001). Therefore, the meta-analysis of the data was performed using the random effects model. The addition of TCM compounds to standard treatments resulted in a beneficial effect on triglyceride levels (MD = −0.46; 95% CI, −0.62 to −0.29; *P*=0.0001; Figure 6).

#### 3.4.6. Low-Density Lipoprotein (LDL-C)

**Figure 7:**
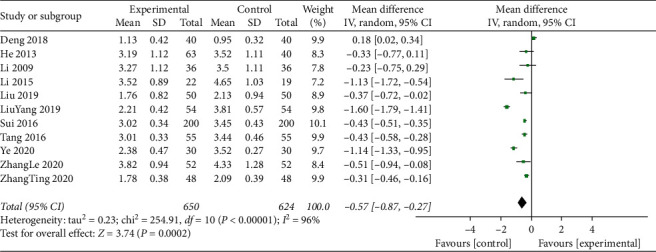
Meta-analysis of low-density lipoprotein cholesterol (LDL-C) levels.

LDL-C levels were reported for a total of 1274 participants in 11studies. These studies had statistically heterogeneous results (*I*^2^ = 96%; *P* < 0.001). Therefore, the meta-analysis of the data was performed using the random effects model. The addition of the effect of TCM compounds to standard treatments resulted in a beneficial effect (MD = −0.57; 95% CI, −0.87 to −0.27; *P*=0.0002; Figure 7).

#### 3.4.7. High-Density Lipoprotein (HDL-C)

**Figure 8:**
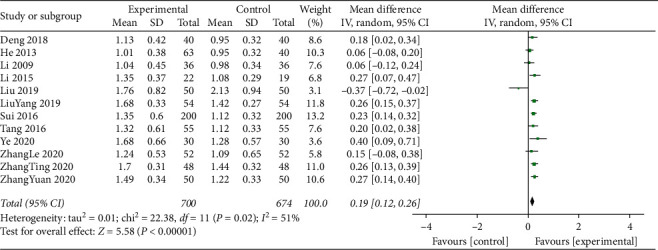
Meta-analysis of high-density lipoprotein cholesterol (HDL-C) levels.

HDL-C levels were reported for a total of 1374 participants in 12 studies were statistically heterogeneous (*I*^2^ = 51%; *P*=0.02). Therefore, the meta-analysis was performed using the random effects model. The addition of TCM compounds to standard treatments resulted in a beneficial effect (MD = 0.19; 95% CI, 0.12 to 0.26; *P*=0.0001; Figure 8).

#### 3.4.8. Effect on Electrocardiogram

**Figure 9:**
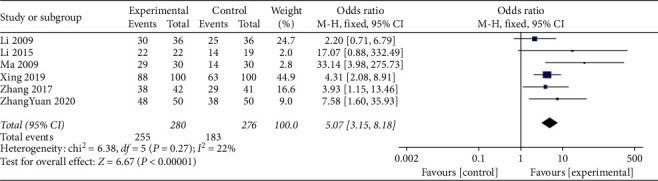
Meta-analysis of the effects on the electrocardiogram.

Electrocardiograms were reported for a total of 556 participants across 6 studies with no statistical between-study heterogeneity (*I*^2^ = 22%; *P*=0.27). Therefore, the meta-analysis of the data was performed using the fixed effect model. The addition of TCM compounds to standard treatments resulted in a beneficial effect on the electrocardiograms (OR = 4.20; 95% CI, 3.15 to 8.18; *P*=0.0001; Figure 9).

#### 3.4.9. Safety Assessment of TCM Compounds

**Figure 10:**
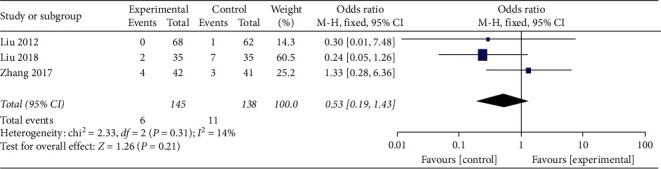
Meta-analysis of adverse reactions.

Safety assessment of TCM compounds was performed in 3 studies with no statistical between-study heterogeneity (*I*^2^ = 14%; *P*=0.31) including a total of 283 participants. A meta-analysis of the data from these three studies was performed using the fixed effect model. The use of TCM compounds combined with standard treatments was not associated with significant difference in the adverse effects recorded (OR = 0.53; 95% CI, 0.19 to 5.50; *P*=0.21; Figure 10).

#### 3.4.10. Publication Bias Analysis

**Figure 11:**
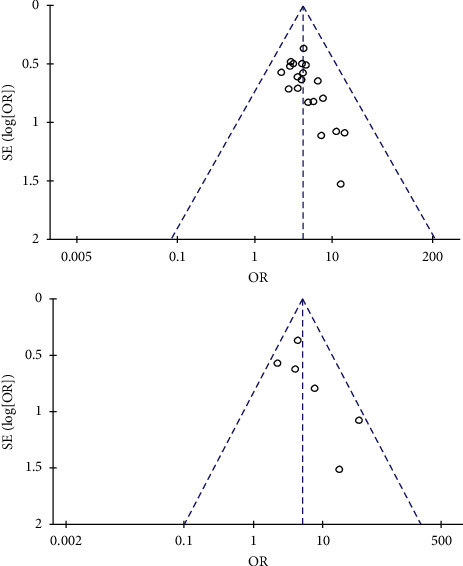
Analysis of publication bias of clinical total effective rate and electrocardiogram effective rate.

Publication bias on the outcome of clinical efficiency and electrocardiograms in the publications included in this study was analyzed according to the pooled qualitative (funnel plot) method. Using the log (OR) as boundary, the studies were asymmetrically distributed between the left- and right-hand sides, indicating a publication bias. (Figure 11).

## 4. Discussion

With lifestyle changes, urbanization and aging of the population, cardiovascular diseases, and cancer have become the leading causes of death in Chinese adults [10]. The prevalence of DM and prediabetes in Chinese adults are 9.7% and 15.5%, respectively. CHD and DM have become major public health problems. There is an interaction between DM and cardiovascular diseases. The risk of cardiovascular events in diabetic patients is nearly twice as high as in nondiabetic patients [36]. Data showed that about 75% of the deaths occurring in DM patients are associated with coronary artery diseases [37]. The results of the European Heart Survey also showed that about two-thirds of the CHD cases are associated with glucose metabolism disorders. Therefore, the treatment of DM complicated by CHD is an important issue.

Standard treatments mostly included oral hypoglycemic drugs or insulin to control blood glucose levels in addition to the management of lifestyle factors, such as diet, exercise, and weight control. However, DM requires long-term continuous control, patient compliance is poor, and the results of these therapeutic strategies are not satisfactory. DM with CHD is classified in the “Xiaoke” and “chest arthralgia” category in TCM theory. The disease is mainly caused by Qi Yin deficiency. Qi and blood stasis caused by the heart pulse are insuperable. Qi deficiency leads to weak blood flow, blood stasis, long-term heat accumulation, and heat injury, provoking further inside blood stasis [38]. Western medicine uses nitrates for the treatment of CHD symptoms, but drug resistance occurs during the treatment of DM, when the use of hypoglycemic drugs increases the load on the heart. The combination of these two types of drugs, which represent an almost life-long treatment, seriously reduces patients' quality of life [39]. Many studies have shown that Chinese herbal compounds can be used to successfully treat CHD. These not only improve clinical efficiency but also relieve FBG, total cholesterol, triglyceride, LDL-C, and 2hPG levels, and other DM indices to some extent.

The meta-analyses we performed showed some limitations of the evaluation system: (1) some publication bias was detected; (2) the overall quality of the included studies was low; (3) there was a lack of objective unified criteria for the determination of clinical efficacy; (4) the control groups received different treatments; and (5) all the studies were published in Chinese.

The results of the analysis could be biased. Although there are some limitations in TCM due to the lack of basic research, evidence-based practices that may be effective make it an attractive treatment system for many diseases. The accuracy of the meta-analysis results depends on the article selection. Therefore, the requirements for future experiments should be multicenter, large sample, randomized, control, and double-blind allocation hidden experiment. These requirements suggest that clinical trials have to further improve their scientific value, strictly control the implementation of standards, provide clearer and more detailed descriptions of randomization, blind test, and more particularly, improve the quality of the testing methodology and standardize the clinical RCT reports, such that they can provide more effective information for clinical applications.

## 5. Conclusion

Of the 23 publications included in this study, 20 reported information on clinical comprehensive efficiency, 11 reported information on FBG levels, 13 reported information on total cholesterol levels, 12 reported information on triglyceride levels, 11 reported information on LDL-C levels, and 11 reported information on HDL-C levels. All of these parameters were statistically improved by the use of TCM compounds.

In summary, based on current evidence, we conclude that combined therapy could increase the clinical total effective rate and reduce FBG, total cholesterol, and HDL-C levels. There was no evidence showing that combination therapy would lead to safety problems. A number of sensitivity analyses have shown that our conclusions are robust.

After evaluating the quality of the articles included in the present meta-analysis, we found that most articles were of low quality, which is the main limitation of our study. International methodologies and rigorous RCTs can produce better tests. Therefore, in order to evaluate the clinical efficacy of TCM and provide a strong scientific basis for the development of TCM, more high-quality studies are needed to provide more reliable data for meta-analysis.

## Figures and Tables

**Table 1:** Basic characteristic of included studies.

Inclusion studies	Sample size		Male/female		Average age		Intervention measures		Treatment course	Outcome indicators
	*T*	*C*	*T*	*C*	*T*	*C*	*T*	*C*		
Zhang 2020 [13]	50	50	24/26	26/24	66.14 ± 2.25	66.28 ± 2.33	Control group + tongxinluo capsule	Metformin	—	(1) (2) (5) (6) (7)
Zhang 2020 [14]	58	58	39/19	37/21	57.05 ± 10.94	56.46 ± 10.37	Control group + guilou xiebai banxia and huangqi xiaoke decoctions	Metformin hydrochloride sustained-release tablets + isosorbide mononitrate tablets	—	(2) (13)
Zhang 2020 [15]	52	52	30/22	28/24	51.6 ± 8.2	50.4 ± 9.5	Control group + danshen ligustrazine injection	Standard western medicine	2 weeks	(1) (7)
Zhang and Zhang 2020 [16]	48	48	30/18	33/15	60.21 ± 15.37	59.87 ± 14.65	Control group + tongxinluo capsule	Isosorbide mononitrate sustained-release tablets + aspirin enteric-coated tablets + metoprolol tartrate + valsartan capsules + atorvastatin	12 weeks	(7) (11)
Ye 2020 [17]	30	30	14/16	15/15	65.7 ± 8.9	65.2 ± 7.6	Control + buyang huanwu decoction	Calcium antagonists + anti-platelet drugs + *β* receptor blockers + nitrate drugs, lipid-regulating drugs + angiotensin converting enzyme inhibitors	3 months	(1) (5) (6) (7) (10)
Xing et al. 2019 [18]	100	100	45/55	42/58	53.01 ± 2.52	51.84 ± 3.15	Control group + tongxinluo, ganlu xiaoke capsule	Isosorbide dinitrate + aspirin enteric-coated tablets + atorvastatin	—	(4) (5) (6) (14)
Guan and Shang 2019 [19]	39	39	24/15	22/17	54.5 ± 2.9	54.8 ± 2.6	Standard western medicine + mai san	Standard western medicine + compound danshen drop pills	2 months	(1) (9) (12)
Liu et al. 2019 [20]	50	50	34/16	32/18	54.18 ± 8.46	53.66 ± 9.12	Control group + shenqi guilou xiebai banxia decoction	Metformin hydrochloride sustained-release tablets + isosorbide mononitrate tablets	45 days	(1) (7) (15)
Jiang 2019 [21]	35	35	18/17	19/16	57.2 ± 7.4	58.9 ± 8.2	Standard western medicine + phlegm and blood stasis decoction	Standard therapy + trimetazidine hydrochloride	4 weeks	(1) (2) (5) (6)
Zuo 2019 [22]	37	37	21/16	23/14	44.15 ± 3.17	43.93 ± 3.04	+ Shengmai San decoction	Danshen dropping pill + compound danshen dropping pill	2 months	(5) (6) (13)
Liu et al. 2019 [23]	54	54	28/26	29/25	67.03 ± 6.12	66.78 ± 5.81	Control group + musk yangxin san	Sodium trimetazidine + isosorbide mononitrate tablets + aspirin enteric-coated tablets	3 month	(1) (5) (6) (7) (8) (16)
Sun et al. 2018 [24]	40	40	21/19	22/18	53.4 ± 1.5	52.5 ± 1.3	Control group + deconstruction of phlegm and removing blood stasis	Standard western medicine	1 month	(1) (5) (6) (17)
Liu et al. 2018 [25]	35	35	21/14	19/16	46.35 ± 2.36	45.32 ± 2.12	Control group + yiqi shufeng tongluo tang	Atorvastatin	—	(1) (3) (13)
Deng 2018 [26]	40	40	21/19	23/17	61.3 ± 5.1	60.1 ± 5.4	Antidiabetic drugs/insulin + long-acting analgesic drugs + ziyin huoxue decoction	Antidiabetic drugs/insulin + long-lasting analgesic drugs + compound danshen dropping pills	1 month	(1) (4) (5) (6) (7) (14)
Zhang 2017 [27]	42	41	23/19	24/17	61.23 ± 12.51	61.42 ± 12.38	Control group + danhong injection	Standard western medicine	2 weeks	(1) (2) (3) (5)
Yan et al. 2017 [28]	35	35	19/16	20/15	41.1 ± 12.9	42.8 ± 13.2	Control group + yiqihua turbid capsule	Antidiabetic drugs, anti-platelet, *β* receptor blockers, statins	3 months	(1) (2) (5) (6) (13)
Sui 2016 [29]	200	200	100/100	98/102	57.49 ± 1.26	57.5 ± 1.25	Antidiabetic drugs/insulin + long-acting analgesic drugs + ziyin huoxue decoction	Antidiabetic drugs/insulin + long-lasting analgesic drugs + danshen dropping pills	1 months	(5) (6) (7)
Tang 2016 [30]	55	55	30/25	32/23	56.15 ± 1.54	57.1 ± 1.36	Control group + ziyin huoxue decoction.	Antidiabetic drugs + insulin + long-lasting analgesic drugs	1 month	(5) (6) (7)
Li and Shang 2015 [31]	19	22	7/12	9/13	59.86 ± 7.44	61.21 ± 7.38	Control group + qishen yiqi dropping pills	Standard western medicine	8 weeks	(1) (2) (5) (6) (7)
He 2013 [32]	40	63	—	—	—	—	Antidiabetic drugs/insulin + long-acting analgesic drugs + ziyin huoxue decoction	Antidiabetic drugs/insulin + long-acting analgesic drugs + guanxin danshen dropping pills	1 month	(1) (4) (5) (6) (7)
Liu 2012 [33]	68	62	46/22	42/20			Conventional treatment + trimetazidine hydrochloride	Standard treatment + phlegm-dispelling and stasis removing decoction		(1) (2) (3) (4)
Li and Yang 2009 [34]	36	36	14/22	11/25	59.5 ± 8.96	57.62 ± 8.76	Antidiabetic drugs/insulin + long-acting analgesic drugs + ziyin huoxue decoction	Antidiabetic drugs/insulin + long-acting analgesic drugs + compound danshen dropping pills	4 weeks	(1) (4) (5) (6) (7)
Ma 2009 [35]	30	30	15/15	15/15	—	—	Control group + yiqi tongluo capsule	Antidiabetic drugs/insulin + isosorbide dinitrate tablets	1 month	(1) (4) (5) (6) (13) (14)

*T*, observation group; *C*, control group; (1), total effective rate; (2), electrocardiogram effective rate; (3), adverse reaction; (4), angina pectoris attack times; (5), blood glucose; (6), 2-hour plasma glucose (2hPG); (7), blood lipid; (8), hemorheological index; (9), reactive protein; (10), interleukin 6; (11), glycosylated hemoglobin; (12), *β*2-microglobulin; (13), TCM syndrome score; (14), angina pectoris pain duration; (15), vascular endothelial function; (16), coagulation function; (17), heart rate.

**Table 2:** Quality of included trials assessment.

Inclusion studies	Randomized approach (2 points)	Double-blind (2 points)	Withdrawal loss (1 point)	Total	Article quality
Zhang 2020 [13]	Random grouping method	Not described	No shedding	1	Low
Zhang 2020 [14]	Not described	Not described	No shedding	1	Low
Zhang 2020 [15]	Random allocation method	Not described	No shedding	1	Low
Zhang and Zhang 2020 [16]	Random digital tables	Not described	No shedding	3	Medium
Ye 2020 [17]	Random digital tables	Not described	No shedding	3	Medium
Xing et al. 2019 [18]	Random digital tables	Not described	No shedding	3	Medium
Guan and Shang 2019 [19]	Random digital tables	Not described	No shedding	3	Medium
Liu et al. 2019 [20]	Not described	Not described	No shedding	1	Low
Jiang 2019 [21]	Not described	Not described	No shedding	1	Low
Zuo 2019 [22]	Not described	Not described	No shedding	1	Low
Liu et al. 2019 [23]	Not described	Not described	No shedding	1	Low
Sun et al. 2018 [24]	Random digital tables	Not described	No shedding	3	Medium
Liu et al. 2018 [25]	Not described	Not described	No shedding	1	Low
Deng 2018 [26]	Not described	Not described	No shedding	1	Low
Zhang 2017 [27]	File sequence	Not described	No shedding	1	Low
Yan et al. 2017 [28]	Random digital tables	Not described	No shedding	3	Medium
Sui 2016 [29]	Not described	Not described	No shedding	1	Low
Tang 2016 [30]	Not described	Not described	No shedding	1	Low
Li and Shang 2015 [31]	Random digital tables	Not described	No shedding	3	Medium
He 2013 [32]	Random digital tables	Not described	No shedding	3	Medium
Liu 2012 [33]	Not described	Not described	No shedding	1	Low
Li and Yang 2009 [34]	Not described	Not described	No shedding	1	Low
Ma 2009 [35]	Not described	Not described	No shedding	1	Low

## Data Availability

All the data generated or analyzed during this study are included in this published article.

## Abbreviations

AE: Adverse event
CHD: Coronary heart disease
DM: Diabetes mellitus
FBG: Fasting blood glucose
HDL-C: High-density lipoprotein-cholesterol
LDL-C: Low-density lipoprotein-cholesterol
MD: Mean difference
OR: Odds ratio
RCT: Randomized controlled trial
TCM: Traditional Chinese medicine
TER: Total efficiency rate
T2DM: Type 2 diabetes mellitus
2Hpg: 2-Hour plasma glucose.
